# Initiation of ART during Early Acute HIV Infection Preserves Mucosal Th17 Function and Reverses HIV-Related Immune Activation

**DOI:** 10.1371/journal.ppat.1004543

**Published:** 2014-12-11

**Authors:** Alexandra Schuetz, Claire Deleage, Irini Sereti, Rungsun Rerknimitr, Nittaya Phanuphak, Yuwadee Phuang-Ngern, Jacob D. Estes, Netanya G. Sandler, Suchada Sukhumvittaya, Mary Marovich, Surat Jongrakthaitae, Siriwat Akapirat, James L. K. Fletscher, Eugene Kroon, Robin Dewar, Rapee Trichavaroj, Nitiya Chomchey, Daniel C. Douek, Robert J. O′Connell, Viseth Ngauy, Merlin L. Robb, Praphan Phanuphak, Nelson L. Michael, Jean-Louis Excler, Jerome H. Kim, Mark S. de Souza, Jintanat Ananworanich, on behalf of the RV254/SEARCH 010 and RV304/SEARCH 013 Study Groups

**Affiliations:** 1 Department of Retrovirology, Armed Forces Research Institute of Medical Sciences – United States Component, Bangkok, Thailand; 2 Henry M. Jackson Foundation for the Advancement of Military Medicine, Bethesda, Maryland, United States of America; 3 AIDS and Cancer Virus Program, Leidos Biomedical Research Inc., Frederick National Laboratory of Cancer Research, Frederick, Maryland, United States of America; 4 Clinical and Molecular Retrovirology Section/Laboratory of Immunoregulation, National Institute of Allergy and Infectious Diseases, National Institutes of Health, Bethesda, Maryland, United States of America; 5 Department of Medicine, Faculty of Medicine, Chulalongkorn University, Bangkok, Thailand; 6 SEARCH, Bangkok, Thailand; 7 The Thai Red Cross AIDS Research Centre, Bangkok, Thailand; 8 Human Immunology Section, Vaccine Research Center, National Institute of Allergy and Infectious Diseases, National Institutes of Health, Bethesda, Maryland, United States of America; 9 U.S. Military HIV Research Program, Walter Reed Army Institute of Research, Silver Spring, Maryland, United States of America; 10 Virus Isolation and Serology Laboratory Applied and Developmental Research Directorate Science Applications International Corporation, Frederick, Inc. National Cancer Institute, Frederick Cancer Research and Development Center, Frederick, Maryland, United States of America; Miller School of Medicine, United States of America

## Abstract

Mucosal Th17 cells play an important role in maintaining gut epithelium integrity and thus prevent microbial translocation. Chronic HIV infection is characterized by mucosal Th17 cell depletion, microbial translocation and subsequent immune-activation, which remain elevated despite antiretroviral therapy (ART) correlating with increased mortality. However, when Th17 depletion occurs following HIV infection is unknown. We analyzed mucosal Th17 cells in 42 acute HIV infection (AHI) subjects (Fiebig (F) stage I-V) with a median duration of infection of 16 days and the short-term impact of early initiation of ART. Th17 cells were defined as IL-17+ CD4+ T cells and their function was assessed by the co-expression of IL-22, IL-2 and IFNγ. While intact during FI/II, depletion of mucosal Th17 cell numbers and function was observed during FIII correlating with local and systemic markers of immune-activation. ART initiated at FI/II prevented loss of Th17 cell numbers and function, while initiation at FIII restored Th17 cell numbers but not their polyfunctionality. Furthermore, early initiation of ART in FI/II fully reversed the initially observed mucosal and systemic immune-activation. In contrast, patients treated later during AHI maintained elevated mucosal and systemic CD8+ T-cell activation post initiation of ART. These data support a loss of Th17 cells at early stages of acute HIV infection, and highlight that studies of ART initiation during early AHI should be further explored to assess the underlying mechanism of mucosal Th17 function preservation.

## Author Summary

Persistent systemic immune activation is a hallmark of chronic HIV infection and an independent predictor of disease progression. The underlying mechanism is not yet completely understood but thought to be associated with the loss of Th17 cells leading to the disruption of the mucosal barrier and subsequent microbial translocation. However, it remains unclear when these events take place in HIV infection, as the only data available to date are from SIV models. We evaluated the kinetics of Th17 depletion, microbial translocation and subsequent immune activation in early acute HIV infection and the effect of early initiated ART on these events. We discovered that a collapse of Th17 cell number and function, accompanied by local and systemic immune activation, occurs already during acute HIV infection. However, early initiation of ART preserved Th17 number and function and fully reversed any initial HIV-related immune activation. These findings argue for the importance of early events during HIV infection setting the stage for chronic immune activation and for early and aggressive treatment during acute HIV infection.

## Introduction

Eradication of HIV infection has not been achieved except under unique circumstances [1], [2]. Given the limitations of antiretroviral therapy (ART) and recent advances in our understanding of HIV persistence with current treatment regimens, there is a growing recognition that a functional cure for HIV infection is both needed and feasible [3]. Despite potent ART, chronic immune activation, inflammation, and immune dysfunction persist, and are likely to have important effects on the size and distribution of the viral reservoir [4] and non-AIDS (or non-infectious) inflammatory related disorders [5].

Acute HIV infection (AHI), defined here as the period between detectable HIV RNA viremia and reactive IgG enzyme immunoassay (EIA) antibody to HIV proteins [6], [7], is marked by peak viremia (>10^6^ copies/mL), the rapid depletion of gastrointestinal CD4+T cells, followed by a deterioration of the mucosal epithelium and increased microbial translocation [8]–[10], which may not be restored despite prolonged ART [11], [12]. In this context, the importance of an IL-17-producing subpopulation of CD4+T cells (Th17 cells) has been emphasized. Th17 cells are depleted in HIV and pathogenic simian immunodeficiency virus (SIV) infection of humans and rhesus macaques, respectively, but are preserved in SIV infection of the natural hosts, sooty mangabeys and African green monkeys [13]–[15]. In addition, Th17 cells are also preserved in HIV-1 infected long-term nonprogressors [16]. Th17 cells are essential for mucosal immunity as they respond to extracellular bacteria and fungi by promoting neutrophil recruitment and produce antimicrobial peptides such as defensin and mucin [17]–[21]. Furthermore, Th17 cells produce IL-22, which enhances epithelial regeneration and, as a possible consequence of their loss, impaired mucosal restoration and subsequent increased intestinal permeability and microbial translocation may occur [22]–[24]. Lower frequencies of Th17 cells in the sigmoid colon of individuals with chronic HIV infection (CHI) correlated with higher plasma lipopolysaccharide (LPS) and were linked to persistent immune activation [25], [26]. Importantly, the level of immune activation in ART-naïve individuals with CHI is the best predictor of HIV disease progression to AIDS [27]. Despite the significant benefits of ART, immune reconstitution in the gut is often incomplete and immune activation may persist [28], [29].

A recent study by Kim et al. has shown that mucosal Th17 function is altered during HIV infection and can serve as an independent predictor of immune activation. While mucosal Th17 cells were rapidly restored under ART, normalization of Th17 function and local and systemic immune activation was much more delayed, emphasizing the importance of strategies to preserve mucosal Th17 function for potential therapeutic benefit [30]. Studies of Th17 cells during early HIV infection are crucial for understanding the timing and impact on gut epithelial barrier dysfunction and damage, but have been hampered due to difficulties identifying AHI and obtaining relevant tissue from human volunteers. Using real-time pooled nucleic acid testing (NAT) and sequential EIA in high risk HIV-seronegative subjects attending voluntary counseling and testing centers (VCT) in Bangkok, we have identified a Thai cohort of volunteers with AHI, mostly infected with HIV-1 circulating recombinant form (CRF) 01_AE and to a lesser extent with subtype B [31], [32] willing to undergo sigmoid biopsies. This cohort represents an unprecedented opportunity to evaluate the impact of AHI on Th17 cells, mucosal barrier integrity and local and systemic inflammation during AHI and moreover allows detailed assessment of the benefit of early initiation of ART on the preservation of CD4+T cell populations and mucosal integrity [33]. Utilizing the unique patient population, we provide the first description of qualitative and quantitative mucosal Th17 cell dynamics, and local and systemic immune activation during the earliest stages of HIV infection in human volunteers. We show that ART initiated during early AHI either prevents loss (Fiebig stage I/II) [6] or restores (Fiebig stage III) mucosal Th17 cells and is consequently associated with normalization of local and systemic immune activation, reversing a hallmark of pathogenic HIV infection. Our study emphasizes the long-term implications of these early events in viral pathogenesis and argues for systematic evaluation of early and aggressive intervention in AHI and evaluation of the underlying mechanism of potential preservation of mucosal Th17 function [34], [35].

## Results

### Characteristics of AHI and chronic HIV-infected subjects

Between May 2009 and March 2012, we identified 42 subjects with AHI, staged according to Fiebig (F) classification at time of HIV diagnosis [6] who were willing to undergo sigmoid biopsy and phlebotomy (S1 Table). Seventeen subjects were identified by pooled nucleic acid test (NAT) (non-reactive HIV IgM antibody – Fiebig I/II) and 25 by sequential EIA (reactive HIV IgM – Fiebig III/IV/V) [6]. Additionally 10 age-, gender- and risk group-matched HIV-uninfected (HIV-) and 5 treatment-naïve subjects with CHI were enrolled to serve as negative and positive controls, respectively. All subjects underwent sigmoid biopsy and phlebotomy. No underlying histopathological findings in the sigmoid colon were observed in our AHI cohort. AHI subjects were mainly young men who have sex with men (MSM) (83%) infected with HIV-1 CRF01_AE (74%) with a median time since history of HIV exposure of 16 days (SD 6.6), a median CD4+T cell count of 465 cells/mm^3^, a median plasma HIV RNA of 5.5 log_10_ copies/mL and a median sigmoid colon HIV RNA of 2.6 log_10_ copies/mg tissue (Table 1). The plasma as well as the colonic HIV RNA increased significantly with progression of infection from FI/II, FIII to FIV/V (plasma HIV RNA: FI/II 4.8 log_10_ copies/ml vs: FIII 6.0 log_10_ copies/ml, p = 0.002, FIV/V 6.2 log_10_ copies/ml, p = 0.02; colonic HIV RNA: FI/II 2.3 log_10_ copies/mg tissue vs: FIII 3.1 log_10_ copies/mg tissue, p = 0.01, FIV/V 3.3 log_10_ copies/mg tissue, p = NS). The mean time elapsed since initial diagnosis of HIV infection for CHI subjects was 298 days (SD 154.1). The median CD4+T cell count was 515 cells/mm^3^ (range 316, 883) and the plasma HIV RNA was 4.9 log_10_ copies/mL (range 4.0, 5.4) (Table 1).

**Table 1:** Clinical, immunological and virological baseline characteristics and demographics of study participants.

Characteristics	Acute HIV-infected (n = 42)	Chronic HIV-infected (n = 5)	HIV-uninfected (n = 10)
Median age [years]	29 (19, 48)^A^	24 (19, 28)^A^	31 (23–41)^A^
Gender Male∶Female	39∶3	5∶0	8∶2
Risk behavior, n (%)			
MSM	35 (83)	5 (100%)	NA
Bisexual male	4 (10)	-	NA
Heterosexual female	3 (7)	-	NA
Fiebig Stage, n			
I/II	17 (13 I, 4 II)	NA	NA
III	21	NA	NA
IV/V	4 (1 IV, 3 V)	NA	NA
Mean (SD) duration of HIV [days]	16 (6.6)	298 (154.1) C	NA
Median plasma HIV RNA [log_10_ copies/ml]	5.5 (2.8, 7.7)^A^	4.9 (4.0–5.4)^A^	NA
Median sigmoid colon HIV RNA [log_10_ copies/mg tissue]	2.6 (1.3, 4.7)^A^	ND	NA
Median CD4 count [cell/mm^3^]	465 (132, 1127)^A^	515 (316, 883)^A^	NA
HIV subtype by MHAbce^B^, n (%) out of 39			
CRF01_AE	29 (74)	ND	NA
B	1 (3)	ND	NA

A range;

B MHAbce: Multi-region hybridization assay distinguishing between subtypes B, C and CRF01_AE. One subject was CRF01_AE/B, 8 were non typable and results for 3 subjects were not typed by the time the manuscript was written;

C Western blot positive with p31 band; MSM: Men who have sex with men; Fiebig I - positive HIV RNA, negative p24 antigen, negative 3rd generation EIA; Fiebig II – positive HIV RNA, positive p24 antigen, negative 3rd generation EIA; Fiebig III - positive HIV RNA, positive p24 antigen, positive 3rd generation EIA, negative western blot; Fiebig IV - positive HIV RNA, positive or negative p24 antigen, positive 3rd generation EIA, indeterminate western blot; Fiebig V - positive HIV RNA, positive p24 antigen, positive 3rd generation EIA, positive western blot except p31; NA: Not Applicable; ND: Not Determined

### Depletion of peripheral blood and colonic CD4+T cells during AHI

Consistent with our previous report [33], the proportion of CD4+T cells in the sigmoid colon significantly decreased with progression of Fiebig stages from a median frequency of 49.8% at FI/II to 35.2% at FIII (p<0.001) and 37.0% at FIV/V (p = 0.009) (Table 2). The same pattern was observed for the proportion of CD4+CCR5+T cells with a decrease from a median frequency of 67.3% in FI/II to 35.5% in FIII (p = 0.002) and 17.4% in FIV/V (p = 0.009). The loss of CD4+CCR5+T cells from FI/II to FIII remained significant when comparing the absolute (abs) numbers (CD4+CCR5+: 6.9×10^6^ cells/g of tissue in FI/II vs. 1.2×10^6^ cells/g of tissue in FIII, p = 0.008 and vs. 0.8×10^6^ cells/g of tissue in FIV/V, p = 0.04). There were no significant differences in the proportion or abs number of CD4+ or CD4+CCR5+T cells between FI and FII. FIII subjects however, showed significant differences when compared to HIV- subjects (%CD4+p<0.001; abs CD4+p = 0.007; %CD4+CCR5+p = 0.002; abs CD4+CCR5+p = 0.002). The observed loss of CD4+CCR5+T cells occurred mainly in the CD27+CD45RO+ central memory (CM) CD4+T cells, and to a lesser extent in the CD27-CD45RO+ effector memory (EM) CD4+T cells (CM: FI/II 48.5%, FIII 18.1%, p = 0.004; EM: FI/II 68%, FIII 32.2%, p<0.001). The absolute numbers of CD8+T cells did not change with progression of Fiebig stages while the frequency increased from 40% in FI/II to 49.4% in FIII (p = 0.009) and to 54.3% in FIV/V (p = 0.007), likely due to losses in CD4+T cells.

**Table 2:** Proportion and absolute number of CD4+, CD4+CCR5+ and CD8+ T cells in sigmoid colon and peripheral blood at baseline in HIV-, FI/II, FIII, FIV/V and CHI subjects.

	FI/II (n = 17)	FIII (n = 21)	FIV/V (n = 4)	HIV- (n = 9)	CHI (n = 5)
**sigmoid colon**					
% CD4+^&^	49.8 (46.4, 58.1)	35.2 (23.8, 43.1)***	37 (24.5, 43.6)**	56.1 (48.9, 61.1)	18.6 (18.4, 28.7)***
absolute CD4+	10 (5, 18)	5 (2, 9)	7 (4, 10)	17 (14, 19)	5 (5, 8)
% CD8+^&^	40 (34.8, 45.4)	49.4 (41.1, 62.7)**	54.3 (49.8, 64.7)**	38 (33, 44.8)	66.3 (58.9, 75.4)***
absolute CD8+	6 (5, 13)	8 (4, 13)	11 (10, 12)	10 (9, 16)	21 (18, 24)
% CD4+CCR5+	67.3 (50.9, 74.4)	35.5 (10.8, 57.5)**	17.4 (10.7, 35.7)**	69.1 (66.5, 70.6)	10.7 (10, 17.8)***
absolute CD4+CCR5+	6.9 (1.8, 12.5)	1.2 (0.2, 4.9)**	0.8 (0.5, 3.6)	11 (9.3, 11.8)	2.3 (0.9, 2.5)
**peripheral blood**					
% CD4+^&^	35 (30, 40)	26 (19, 29)***	21 (15, 25)**	53.9 (51.6, 62.2)***	18 (18, 23)**
absolute CD4+	547 (399, 618)	389 (341, 532)	362 (285, 561)	ND	515 (426, 581)
% CD8+^&^	28 (25, 31)	44 (38, 51)***	56 (49, 61)**	31.5 (29.8, 35.7)*	43 (36, 46)**
absolute CD8+	380 (238, 448)	622 (497, 1000)	1262 (889, 1465)	ND	1166 (1162, 1260)**
% CD4+CCR5+	10.3 (7.9, 13.1)	12.6 (10.4, 16.8)	9.9 (7.9, 15.1)	16.1 (11.9, 18.3)	8 (6, 9.4)
absolute CD4+CCR5+	ND	ND	ND	ND	ND

All data are median (interquartile rang); All comparisons were made to FI/II; *p≤0.05, **p≤0.01 and ***p≤0.001; CHI: Chronically HIV-infected patients; ND: Not Determined; abs numbers in the sigmoid colon are shown as 10^6^ cells per gram tissue and in the peripheral blood as cells/mm^3; &^Percentage of CD4+ and CD8+T cells based on population of CD3+T cells.

In peripheral blood, the proportion of CD4+T cells showed a significant decrease from a median frequency of 35% in FI/II to 26% in FIII (p = 0.003) and to 21% in FIV/V (p = 0.001). FI/II showed a significant decrease of CD4+T cells frequency compared to HIV- (53.9% vs. 35%, p<0.001, respectively) possibly due to redistribution of T-cell subsets. There were no changes seen in the frequency of CD4+CCR5+T cells in peripheral blood. However, a strong correlation was observed between sigmoid colon and peripheral blood for the frequencies of both CD4+T cells and CD4+CCR5+T cells (r = 0.64, p<0.001 and r = 0.73, p<0.001). In contrast, the proportion and abs numbers of CD8+T cells in peripheral blood increased with progression from FI/II to FIII ((%CD8+p<0.001; abs CD8+p = 0.004) (Table 2).

### Depletion of mucosal CD4+T cells during AHI occurs in the lamina propria (LP)

**Figure 1:**
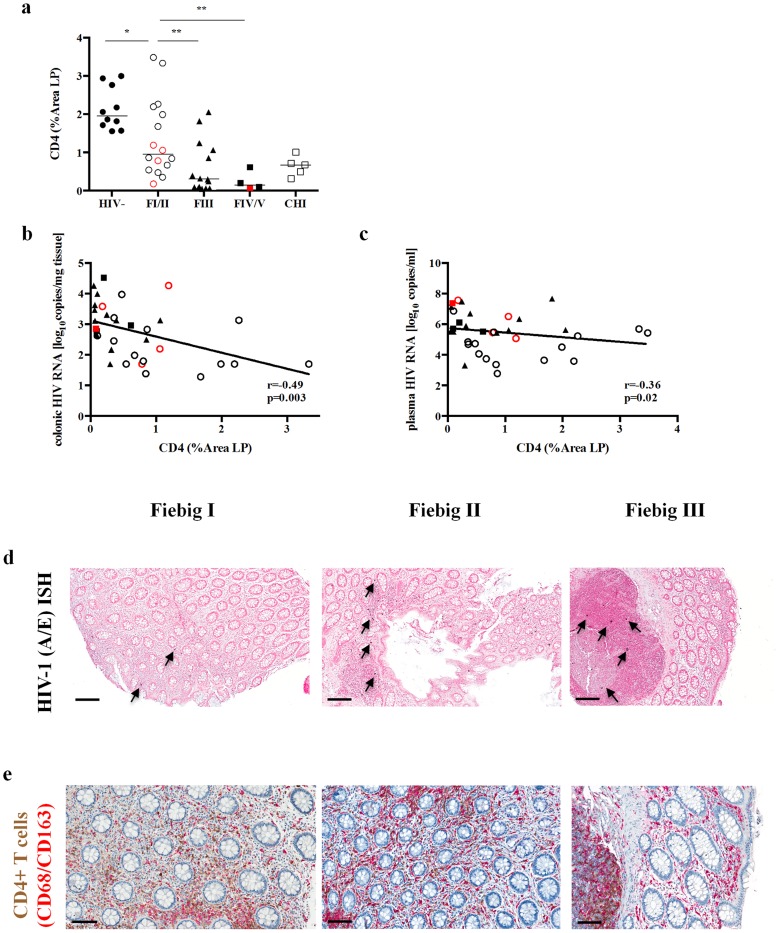
Percentage area of lamina propria CD4+ staining (% Area LP) decreases with progression of Fiebig stage. (**a**) The percent area of the lamina propria that stained for CD4+T cells (representative images shown in e) decreases by Fiebig stage in the sigmoid colon when compared to FI/II (*p≤0.05, **p≤0.01 and ***p≤0.001). The percent area of the lamina propria that stained for CD4+T cells correlated inversely with the colonic (**b**) and plasma (**c**) HIV RNA in FI/II, FIII and FIV/V. HIV viral replication during different Fiebig stages is shown by in *situ hybridization* displaying HIV-1 vRNA+ cells within the sigmoid mucosa, indicated by blue/black stained cells in nuclear fast red counterstained tissue sections (black arrows highlighting examples of HIV vRNA+ cells) of patients in FI, FII and FIII (**d**). CD4+T cell depletion is shown by immunohistochemical staining of CD4+T cells (brown) and macrophages (red) in sigmoid mucosa of patients in FI, FII and FIII (**e**). FI (black circle)/FII (red circle) and FIV (red square)/FV (black square).

In order to localize the depletion of CD4+T cells in the sigmoid colon, immuno-histochemistry and quantitative image analysis was performed to determine the percentage area of LP staining for CD4+T cells (% area LP CD4+). Comparable to the proportion and abs number of CD4+T cells, a significant decrease of the CD4+T cells within the LP was observed (0.96% area in FI/II to 0.31% in FIII (p<0.004) to 0.15% in FIV/V; p = 0.007). In contrast to the overall frequency and abs number of bulk CD4+T cells in the sigmoid colon, the CD4+T cells within the LP decreased between HIV- and FI/II (% area LP CD4+: 1.96 vs. 0.96, p = 0.004, respectively) (Fig. 1a). During AHI an inverse correlation was observed between the % area LP CD4+ and the colonic HIV RNA (r = −0.49, p = 0.003, Fig. 1b) as well as the plasma HIV RNA, which showed a statistically significant, albeit weak correlation (r = 0.36 and p = 0.02; Fig. 1c). The anatomical location of productively infected HIV RNA+ cells (Fig. 1d) was directly related to CD4+T cell populations (Fig. 1e) within the gastrointestinal anatomical compartments (i.e. LP and isolated lymphoid aggregates [LA]). Some patients in FI/II, which demonstrated a variable degree of CD4+T cell depletion within the LP, having had HIV RNA+ cells in both the LP and LA, whereas in FIII concomitant with near complete depletion of CD4+T cells from the LP HIV RNA+ cells are absent from the LP and restricted to the LA where abundant CD4+T cell populations persist. The decrease of CD4+ within the LP correlated with the proportion of CD4+ (r = 0.63 p<0.001) and CD4+CCR5+T cells (r = 0.68 p<0.001) in the sigmoid colon and with the proportion of CD4+T cells in the peripheral blood (r = 0.60 p<0.001).

### Mucosal Th17 cells are depleted early during AHI and show reduced functional capabilities

**Figure 2:**
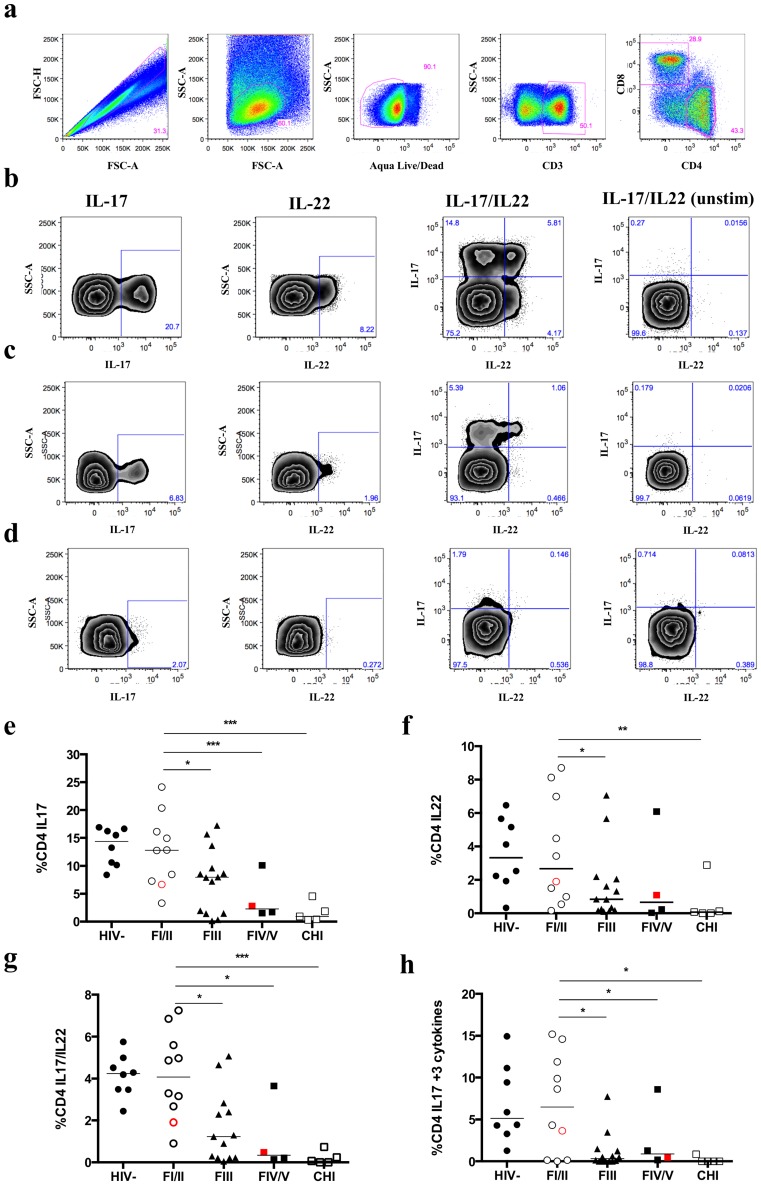
Frequency of IL-17 and/or IL-22 expressing mucosal CD4+T cells decreases by progression of Fiebig stage. To quantify the expression of Il-17 and IL-22 in CD4+T cells, mucosal and peripheral mononuclear cells were stimulated for 5 hours with 40 ng/mL PMA and 1 µM Ionomycin. Gating strategy of sigmoid colon Th17 and Th22 CD4+T cells is shown for a FI subject (**a**) and representative flow cytometry plots including unstimulated controls (unstim) gated on CD4+T cells showing expression of IL-17 and/or IL-22 in FI (**b**), FIII (**c**) and FV (**d**) in the sigmoid colon. A decrease in the frequency of IL-17 (**e**), IL-22 (**f**), IL-17/IL-22 (**g**)-producing cells was seen as well as in the subpopulation of triple-cytokine producing (IL-2, IL-22, IFNγ) Th17 cells (**h**) from FI (black circle)/FII (red circle) to FIII and FIV (red square)/FV (black square). Frequency of single and double cytokine producing cells is calculated from percentage CD4+T cells, while triple-cytokine producing cells are calculated from percentage of CD4+IL17+T cells. All comparisons were made to FI/II: *p≤0.05, **p≤0.01 and ***p≤0.001.

Studies in the pathogenic non-human primate model suggest that mucosal immune dysfunction is attributed to the preferential loss of CD4+ Th17 cells [14], [15], [18]. Recent studies have demonstrated the importance of IL-22 expressing CD4+T cells (Th22) contributing to the homeostasis of mucosal epithelial surfaces [19], [20]. We performed flow cytometric analysis of IL-17- and IL-22-producing CD4+T cells from sigmoid tissue and peripheral blood after stimulation with PMA and ionomycin. Fig. 2 (a to d) shows the gating strategy and representative flow cytometry plots for IL-17 and/or IL-22 expression in different Fiebig stages. Due to limited availability of mucosal mononuclear cells (MMC), data are only available for a subset of subjects: HIV- (8), FI (9), FII (1), FIII (14), FIV (1), FV (3) and CHI (5). IL-17- and IL-22-producing CD4 T cells decreased with progression of Fiebig stages. The proportion of Th17 cells decreased from 12.8% in FI/II to 7.9% in FIII (p = 0.02) and to 2.3% in FIV/V (p = 0.001), and further decreased to 0.9% in CHI (p<0.001, Fig. 2e). The same trend was seen in Th22 cells: 2.9% in FI/II to 1.3% in FIII (p = 0.02) and to 0.4% in FIV/V (p = NS), and in CD4+T cells producing both IL-17 and IL-22 (FIII 1.7%, p = 0.02 and FIV/V 0.9%, p = 0.04 vs. FI/II 3.7%, Fig. 2f). The proportion of IL-17- and/or IL-22-producing CD4+T-cell subsets in FI/II was comparable to HIV- subjects (Th17: 12.8% vs. 13.5%, p = NS; Th22: 4.1% vs. 3.6%, p = NS; IL-17/IL-22: 4.1 vs. 4.0, p = NS, respectively, Fig. 2g). There was a significantly lower proportion of Th17 cells in peripheral blood (HIV-: 0.37%, FI/II: 0.65%, FIII: 0.5% and FIV/V: 0.86%, CHI: 0.48%) compared to the sigmoid colon with no quantitative changes observed between Fiebig stages. Th22 cells were not detected in the peripheral blood. In the sigmoid colon, the frequencies of IL-17, IL22 and IL-17/IL-22-producing CD4+T cells in AHI were correlated with the frequency of bulk CD4+T cells (r = 0.67, p<0.0001; r = 0.59, p = 0.001 and r = 0.80, p<0.0001, respectively) and the % area LP CD4+ (r = 0.71, p<0.0001: r = 0.52, p = 0.005 and r = 0.64, p = 0.0003, respectively). In addition, the frequencies of IL-17 and IL-17/IL-22-producing CD4+T cells were inversely correlated with colonic HIV RNA (r = −0.58, p = 0.003 and r = −0.30, p = 0.03, respectively). A recent study showed that not only the frequency, but also the function of mucosal Th17 cells is altered during HIV infection [30]. Therefore, we assessed the polyfunctionality of Th17 cells by the co-expression of IFN-γ, IL-2 and/or IL-22 using Boolean gating. We observed a dramatic loss of the triple cytokine-producing subset of Th17 cells from FI/II (6.5%) to FIII (0.3%, p = 0.02) and to FIV/V (0.7%, p = 0.03) correlating with the loss of bulk CD4+T cells (r = 0.47, p = 0.01) and % area LP CD4+ (r = 0.45, p = 0.02). This population of polyfunctional Th17 cells was entirely depleted in CHI (Fig. 2h).

In the context of Th17 depletion and the integrity of the mucosal barrier, several studies have shown that CD4+CD25+FoxP3+ regulatory T cells (Treg) exert anti-inflammatory functions and control self-reactive T cells, including Th17 cells [36], [37]. Therefore the frequency of Treg was measured in the sigmoid colon. No significant difference in the percentage of Treg was observed throughout Fiebig stages compared to HIV-, but as described earlier [38] CHI displayed a significantly higher frequency of Treg compared to HIV- and AHI subjects (HIV-: 8.5%, FI/II: 7.5%, FIII: 7.7%, FIV/V: 8% vs. CHI: 13.1%, p<0.001). However, an increase in the frequency of cycling Treg (Ki67+) was observed with progression of Fiebig stages from 5.6% in FI/II to 7.1% in FIII (p = NS) and to 14.7% in FIV/V (p = 0.03; S1 Figure). An inverse correlation between the proportion of cycling Treg and Th17 cells supports the hypothesis of an early host counter-regulatory response to local/systemic inflammation and immune activation, in part, due to the loss of Th17 cells and disruption of the mucosal epithelium early in AHI (r = −0.66, p<0.001).

### Loss of Th17 cells early in AHI is associated with local and systemic immune activation

In order to assess whether the loss of Th17 cells impacts microbial translocation and immune activation, we determined the plasma levels of different biomarkers and assessed the activation of CD4+ and CD8+T cells in the sigmoid colon and the peripheral blood. The proportion of mucosal Th17 cells in AHI subjects showed an inverse correlation with plasma levels of C-reactive protein (CRP; r = −0.42, p = 0.03), Hyaluronic Acid (HA; r = −0.53, p = 0.003), TNFα (r = −0.49, p = 0.03) and IP-10 (r = −0.71, p<0.001), indicating that the loss of Th17 cells might contribute to microbial translocation that leads to the observed levels of systemic immune activation. No correlations were seen between the frequency of Th17 cells and biomarkers that indicate intestinal damage (I-FABP), microbial translocation (LPS and sCD14), and activation of the coagulation cascade (D-dimer) (S2 Figure).

Next, we determined T-cell activation in the sigmoid colon and the peripheral blood by measuring the frequency of HLA-DR and CD38 co-expression on CD4+ and CD8+T cells (Table 3). CD4+T-cell activation in the sigmoid colon increased from 1.4% in HIV- to 2.0% in FI/II (p = 0.03) and to 2.7% in FIII (p = 0.02), while there was no statistically significant increase seen in the peripheral blood of these patients. CD8+T-cell activation significantly increased with Fiebig stage in the sigmoid colon (FI/II 4.4% vs. FIII 8.9%, p = 0.003) and the peripheral blood (FI/II 7.8% vs. FIII 15%, p = 0.004). FI/II subjects had higher CD8+T-cell activation compared to HIV- subjects in the sigmoid colon (4.4% vs. 1.9%, p<0.001, respectively) and the peripheral blood (7.8% vs. 3.0%, p<0.001, respectively). A similar increase was observed for cycling CD8+T cells (Ki67 positive) in the sigmoid colon and the peripheral blood from 4.3% and 5.4% in FI/II to 14.6% and 9.0% in FIII (p<0.001 and p = 0.01), respectively, as well as cycling CD4+T cells in the sigmoid colon (FI/II: 1.8%, FIII: 3.0%, p = 0.04). Cycling CD4+ and CD8+T cells in the sigmoid colon and the peripheral blood of AHI subjects correlated inversely with the frequency of CD4+T cells in the respective compartments. This correlation could only be observed for activated CD4+T cells in the peripheral blood and activated CD8+T cells in the sigmoid colon. Cycling CD4+ and CD8+T cells in the sigmoid colon and cycling CD8+T cells in the peripheral blood correlated with plasma and colonic HIV RNA while only activated CD8+T cells in the colon correlated with plasma and colonic HIV RNA (S2 Table). The frequency of mucosal Th17 cells correlated inversely with the proportion of activated CD8+T cells in the periphery and the sigmoid colon r = −0.43, p = 0.02 and r = −0.40, p = 0.03 respectively) and cycling CD8+T cells (r = −0.51, p = 0.005 and r = −0.56, p = 0.001 respectively), suggesting that the loss of Th17 cells at the mucosa is associated with local and systemic immune activation early in AHI.

**Table 3:** Percentage of activated (HLA-DR+CD38+) and cycling (Ki67+) CD4+ and CD8+ T cells in sigmoid colon and peripheral blood at baseline in HIV-, FI/II, FIII, FIV/V and CHI subjects.

		FI/II (n = 17)	FIII (n = 21)	FIV/V (n = 4)	HIV- (n = 9)	CHI (n = 5)
**sigmoid colon**						
**% CD4**	DR+CD38+	2.0 (0.6, 5.4)	2.7 (1.1, 5.5)*	2.5 (1.0, 6.0)	1.4 (3.3, 2.1)*	3.6 (3.0, 8.5)**
	Ki67+	1.8 (0, 12.8)	3.0 (0.6, 15.2)*	6.6 (3.7, 12.3)*	1.0 (0.7, 2.5)*	9.4 (4.4, 11.1)**
**% CD8**	DR+CD38+	4.4 (1.1, 24.8)	8.9 (3.8, 40.2)**	11.5 (8.5, 11.5)	1.9 (0.9, 1.9)***	7.6 (6.5, 26.2)***
	Ki67%	4.3 (1.2, 37.9)	14.6 (3.0, 64.4)***	14.4 (11, 21.1)**	2.9 (0.8, 9.6)*	4.9 (3.5, 17.7)
**peripheral blood**						
**% CD4**	DR+CD38+	2.0 (0.1, 4.2)	2.6 (0.5, 6.9)	3.4 (2.1, 5.1)	1.5 (0.4, 3.3)	2.1 (1.8, 3.0)
	Ki67+	1.7 (0.4, 4.5)	1.9 (1.3, 3.2)	2.5 (1.3, 3.2)	0.8 (0.2, 1.3)***	1.5 (1.2, 1.8)
**% CD8**	DR+CD38+	7.8 (1.9, 20.5)	15 (8.3, 54.4)**	15.6 (5.0, 32)	3.0 (0.1, 2.9)***	17.8 (12.1, 23.8)*
	Ki67%	5.4 (0.7, 9.5)	9.0 (0.5, 34)***	20.4 (4.1, 25.5)**	2.5 (0, 4.9)**	3.0 (1.7, 4.6)

All data are median (interquartile range); All comparisons were made to FI/II: *p≤0.05, **p≤0.01 and ***p≤0.001; CHI: Chronically HIV-infected patients; DR: HLA-DR.

### Early initiation of ART prevents loss of mucosal Th17 cells, preserves their functional status and normalizes systemic and local immune activation

**Figure 3:**
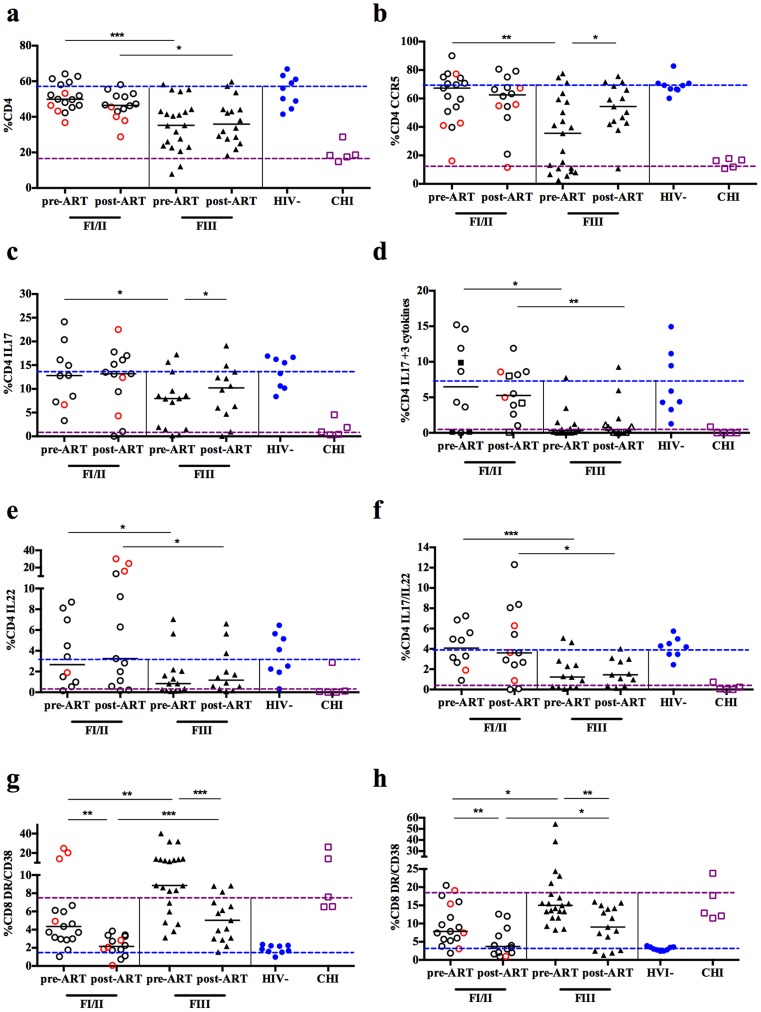
Impact of early ART initiation on CD4+ and CD8+T cells as well as Th17 cells. Subjects that initiated ART during FI (black circle)/FII (red circle) for 6 months were able to maintain mucosal CD4+T cells (**a**), CD4+CCR5+ (**b**), Th17 cells (**c**), triple cytokine-producing Th17 cells (**d**), Th22 cells (**e**), and IL-17 and/or IL-22 (**f**) producing CD4+T cells with no differences when compared to HIV-. In addition their CD8 activation in the mucosa (**g**) and periphery (**h**) normalized after 6 months of ART. Frequency of single and double cytokine producing cells was calculated from percentage CD4+T cells, while triple-cytokine producing cells were calculated from percentage of CD4+IL17+T cells. *p≤0.05, **p≤0.01 and ***p≤0.001; DR: HLA-DR; blue dotted line: median of HIV- individuals; purple dotted line: median of CHI individuals.

Patients identified during early AHI were immediately placed onto ART and these subjects were followed for 6 months to observe the short-term effect of early initiation of ART. Six months post-ART 29 subjects underwent sigmoid biopsy and phlebotomy, of whom 14 were FI/II and 15 were FIII. AHI subjects had an increased median peripheral blood CD4+T cell count at 6 months of 611 cells/mm^3^ compared to 465 cells/mm^3^ at pre-ART (p<0.001). All subjects had undetectable plasma HIV RNA and colonic HIV RNA was undetectable in 28/29 subjects. In addition, after 6 months of ART no subjects had any evidence of HIV vRNA+ cells in colonic tissues as measured by *in situ* hybridization. The proportion of mucosal CD4+ and CD4+ CCR5+T cells remained stable between pre- and post-ART in patients treated at FI/II: 49.8% vs. 46.5% and 67.3% vs. 62.5%, respectively (p = NS). Subjects treated at FIII, who had significantly lower frequencies of CD4+ and CD4+CCR5+T cells pre-ART compared to patients in FI/II, showed an increased frequency of CD4+CCR5+T cells (35.5% pre-ART vs. 54.5% post-ART, p = 0.02), but not CD4+T cells (35.2% pre-ART vs. 35.9% post-ART, p = NS; Fig. 3a and b). Subjects treated at FIII demonstrated a significant decrease in plasma levels of CRP from 1343 pg/ml to 483 pg/ml (p = 0.02) and D-dimer from 359 pg/ml to 146 pg/ml (p<0.001) from pre- to post-ART, respectively.

Subjects treated during FI/II maintained polyfunctional Th17 cells, with no loss of either total Th17 cells or the proportion of triple cytokine-producing Th17 cells post-ART. Those treated during FIII showed a complete restoration of total Th17 cells (7.9% pre-ART vs. 10.2% post-ART, p = 0.05); however short-term ART did not restore the population of triple cytokine-producing Th17 cells which remained at levels comparable to CHI subjects (Fig. 3c and d). Similar observations were made for Th22 cells and cells expressing IL-17 and/or IL-22. While subjects treated at FI/II maintained frequencies of these cell populations at levels similar to HIV- individuals, those treated at FIII did not recover these cell populations (Fig. 3e and f).

Because short-term ART initiated during FIII did not reconstitute mucosal Th17 cell function, but reduced plasma levels of CRP and D-dimer, which are associated with mortality [39], we also explored the impact of ART initiated early in AHI on local and systemic immune activation (Fig. 3g and h). Pre-ART initiation, there was a higher frequency of activated (HLA-DR+/CD38+) CD8+T cells in FI/II (p≤0.001) and FIII subjects (p≤0.0001) after 6 months of ART compared to HIV- in both the sigmoid colon and the peripheral blood (Table 3). This increased activation was more marked in subjects treated in FIII compared to those treated in FI/II. In both the sigmoid colon and the peripheral blood, a decrease of activated CD8+T cells was seen in FI/II and FIII treated subjects. Only those subjects treated in FI/II demonstrated a normalization of CD8+T cell activation levels following short-term ART (sigmoid colon: 2.1% post-ART vs. 4.4% pre-ART, p = 0.001 vs. 1.9% HIV-, p = NS; peripheral blood: 3.7% post-ART vs. 7.8% pre-ART, p = 0.007 vs. 3.0% HIV-, p = NS). Subjects treated during FIII failed to normalize CD8+T-cell activation and showed significantly higher activation compared to HIV- individuals (sigmoid colon: 5.0% post-ART vs. 8.9% pre-ART, p≤0.001 vs. 1.9% HIV-, p≤0.001; peripheral blood: 9.0% post-ART vs. 15% pre-ART, p = 0.003 vs. 3.0% HIV-, p≤0.001, S3, S4 and S5 Tables).

## Discussion

Progressive HIV infection is characterized by a rapid depletion of gastrointestinal CD4+T cells, with a preferential loss of mucosal CD4+ Th17 cells, which play an important role in maintaining intestinal integrity [10], [14], [25]. However, defining the timing of these events during the earliest stages of AHI and determining the impact of early acute ART initiation on Th17 cell loss and recovery have not been determined. We described cellular events in the earliest stages of acute HIV-1 infection confirming and extending previous findings from the pathogenic non-human primate (NHP) model that suggest early disappearance of mucosal Th17 cells contributes to deterioration of the mucosal barrier and subsequent systemic immune activation [15], [25]. While CD4+T cells in peripheral blood recover after AHI, reconstitution of mucosal CD4+T cells is only partial under ART and, in the case of Th17 cells, functional restoration is much delayed [30]. We demonstrate here that initiation of ART prior to HIV IgM detection (Fiebig I/II) prevented the functional and quantitative loss of mucosal Th17 cells as well as a normalization of local and systemic T-cell activation.

Concordant with previous NHP studies [40], [41] we observed a massive depletion of CD4+ and CD4+CCR5+T cells in the colon within days of HIV-1 infection correlating with the colonic HIV RNA. Initiation of ART in FI/II prevented CD4+T cell loss over the course of the first 6 months of treatment, while initiation of ART in FIII only partially restored the frequency of CD4+CCR5+T cells and did not restore CD4+T cells. This supports the hypothesis that initial damage to the mucosal CD4+ T-cell compartment cannot be fully restored even after long term ART [28]. However, we observed a more drastic CD4+T-cell depletion in the LP, the main effector site in the GI tract [42], which showed a significant loss of CD4+T cells as early as FI/II stages. The discrepancy between immunohistochemistry and the bulk CD4+T cells assessed by flow cytometry is potentially methodological. There might be a less dramatic loss occurring in the lymphoid aggregates (LA), and the bulk CD4+T cells in the sigmoid digests do not allow discrimination between LA and LP. In addition, LA are more likely to be observed with high numbers of biopsies as collected for flow cytomerty of bulk CD4+T cells (20 to 25 pieces). Due to small numbers of biopsies dedicated to immunohistochemistry assessment (1 to 2 pieces), very few to no LA were identified within a given biopsy, which possibly accounts for the more dramatic CD4+T-cell loss seen in the histological assessment. In addition, our findings within the sigmoid colon may not reflect the distribution of CD4+T cells within other portions of the gastrointestinal tract, such as ileum or duodenum [43]. Previous studies have reported that CD4+T-cell depletion is more severe in the duodenum [44], ileum [45], [46], and colon [28] than in the blood of treatment-naïve patients and in the duodenum, compared with the colon and rectum [47] of patients receiving suppressive ART.

Mucosal Th17 cells express a wide range of functions compared to those in blood including the production of IL-22, IL-17A, IL-17F, IL-1, IL-2 and IL-21 that together induce the expression of defensins and other antibacterial products [48]. In addition they also produce several effector functions such as TNFα and IFNγ to recruit neutrophils and myeloid cells to effector sites by inducing granulocyte macrophage colony-stimulating factor, and are involved in the regeneration of mucosal epithelium [49]-[51]. During acute SIV infection, the frequency of Th17 cells at mucosal sites decreases dramatically and is not restored to normal levels at the chronic phase [18]. The mechanism related to the apparent loss of Th17 cells is not completely understood, but might be due to the fact that these cells are highly activated because of continuous exposure to bacterial antigens. Th17 cells also express CCR5 and α4β7 and therefore might become a preferential target for HIV-1 [52]. A recent study in humans suggests that Th17 cells are partially restored after ART but the recovery of Th17 function was dramatically delayed [30]. The current study examined evolution and function of mucosal Th17 cells and their relationship with microbial translocation and local and systemic immune activation in early AHI. We demonstrated, for the first time in humans, that Th17 cells are already depleted by FIII, while being preserved during FI/II stages compared to HIV- individuals. The events that lead to this rapid loss of Th17 cells in AHI are not well understood. CD4+T-cells loss has previously been linked mainly to apoptosis [53], [54]. However, recent studies have suggested that pyroptosis, a highly inflammatory form of programmed cell death, in which dying cells release their cytoplasmic contents and inflammatory cytokines into the extracellular space is a potential mechanism of HIV-related CD4+T-cell death [55]. This cell death pathway thus links the two signature events in HIV infection, CD4 T-cell depletion and chronic inflammation, which might explain the high levels of local and systemic immune activation observed in this study.

We also observed a depletion of Th22 cells in FIII, a subset of mucosal CD4+T cells that provides innate immune protection against bacterial and fungal infections and promote inflammation and epithelial proliferation and repair by secretion of IL-22 [56]–[58] and Th17 cells co-expressing IL-22. The frequencies of mucosal Th17 and Th22 cells were inversely correlated with colonic HIV RNA, supporting the relation between the loss of Th17 T cells and viral replication in early AHI [52]. In parallel with the loss of mucosal Th17 cells we also observed a decrease in polyfunctionality (defined here as IL-17-expressing CD4+T cells that co-express IL-2, IL-22 and IFNγ). Triple cytokine-producing Th17 cells were present during FI/II at levels comparable to HIV- individuals but were depleted as early as FIII and were absent in treatment-naïve CHI subjects. ART initiated during FI/II was able to completely preserve Th17 cell numbers and polyfunctionality as well as Th22 cells, while ART initiation in Fiebig III or later only partially restored the Th17 cell numbers but did not restore polyfunctionality or Th22 cell numbers. These findings highlight that events during very early AHI may have long-term consequences in viral pathogenesis not reflected in peripheral blood and that even though Th17 numbers can be restored under ART, Th17 function remains impaired and, may still mediate microbial translocation and immune activation [25].

During AHI we did not observe an increase in mucosal Treg, which may also contribute to the disruption of the mucosal barrier [59]. In HIV and SIV infection, Treg may decrease chronic immune activation, thereby slowing disease progression [60], but potentially may also inhibit anti-viral immune responses, thereby accelerating disease progression [36], [61]–[64]. We hypothesize that these events occur later during HIV infection, as previous studies have shown that non-controllers have significantly higher percentages of Treg in rectal MMCs compared to HIV- individuals or HIV elite controllers [38]. In addition, the frequency of cycling (Ki67+) Treg increased with Fiebig stages, and inversely correlated with the frequency of Th17 cells. NHP studies have shown that cycling Treg increase at later time points following infection and correlate with immune activation in the LP [15], implying that dysregulation of Treg begins at early AHI and eventually progresses to the potentially harmful increase in Treg seen in CHI [36], [61]–[64].

The observed decrease in Th17 cells with the progression of Fiebig stages was directly associated with local and systemic immune activation. Thus, the observed loss of Th17 cells might initiate a vicious cycle early in HIV infection by decreasing the host defense to bacteria that may favor breaches in the GI barrier and result in further increase in local as well as systemic immune activation [65]. T-cell immune activation has been linked to AIDS and non-AIDS related morbidity and mortality in untreated HIV infection [66]. In patients receiving ART, higher T-cell activation has been linked to diminished CD4+T-cell recovery [67], and surrogate markers of cardiovascular disease and increased mortality [68]. In AHI we observed increased local and systemic T-cell activation and cycling with progression of Fiebig stage, compared to HIV- individuals. This seems to be most profound in the CD8+T-cell compartment beginning as early as FI/II. The increase in cycling CD4+ and CD8+T cells in both compartments was directly related to the depletion of CD4+T cells at the respective site, suggesting that during early AHI a similar mechanism induces CD4+ T-cell depletion and increased cycling of CD4+ and CD8+ T cells in blood and sigmoid mucosa. This observation supports earlier findings that there might not be a compartmentalization between these two distinct sites [46].

The loss of Th17 cells correlated inversely with local and systemic T-cell activation and CRP and HA plasma levels, which are increased in treatment-naïve CHI and associated with an increased risk of developing AIDS [69]. More importantly, the local and systemic CD8+T-cell activation was significantly lower in FI/II compared to FIII and normalized after initiation of ART in FI/II to levels comparable to HIV- individuals. Subjects treated in FIII also showed a significant reduction of CD8+ T-cell activation, which did not normalize to that observed in HIV- individuals. We hypothesize that this might be due either to the greater size of the persistent HIV reservoir in FIII compared to FI/II, as cellular reservoirs of latent integrated HIV are established quickly after infection, or to ongoing low replication levels [70]. Previous studies have shown that initiation of ART less than 6 months after HIV infection was able to decrease chronic CD8+ T-cell activation and limit the size of the persistent reservoir [71], [72]. During long-term ART, residual T-cell activation and inflammation consistently correlate with disease progression, but the ability of early ART to prevent these potentially irreversible outcomes remains unclear [67], [73]. Initiation of ART within 6 months of infection compared to later reduces the CD8+ T-cell activation, but levels remained elevated compared to HIV- individuals [71]. However, there was a significant decrease of systemic and local CD8+ T-cell activation in FIII at 6 months post-ART initiation. We cannot exclude that those values will normalize after long-term ART, taking into account that there were much higher levels at the time of diagnosis in FIII compared to FI/II.

Firstly, while the cohort is unique with regard to the time of diagnosis and specimen sampling, our study has some limitations. The challenge of recruiting subjects early during AHI has resulted in a relatively small sample size and as the mucosal biopsies at each visit were entirely optional, matched consecutive samples were not available from all volunteers. In addition there is an inherent inter-individual variability to mucosal sampling. In addition, the sample size for CHI is low and while this group showed significant differences to AHI subjects, we did not assess differences in comparison to HIV+ individuals that initiated ART in the chronic phase of infection, a clinically more relevant population. Our study cannot define the mechanism leading to the loss of mucosal Th17 cells and their functional capabilities. We speculate that polyfunctional mucosal Th17 cells might be more susceptible to HIV infection due to a higher activation status because of continuous exposure to bacterial antigens [14] and therefore are profoundly depleted with progression of Fiebig stage. In addition, slower HIV clearance in the gut mucosa after ART initiation may hinder or delay functional reconstitution of Th17 cells [74], which was not observed after short-term ART in patients initiating treatment in FIII. The factors contributing to the depletion and restoration of polyfunctional Th17 cells and their potential role in tissue viral reservoirs will be an important area of further research. In addition, our experimental design focused on the expression of IL-17, IL-2, IL-22 and IFN-γ, leaving the likely possibility that HIV also alters production of other cytokines secreted by Th17 cells. Furthermore, despite observing local and systemic immune activation during early AHI, we did not find an association between the loss of Th17 cells and plasma biomarkers of microbial translocation. *In vivo* studies have shown that microbial translocation results from a series of immunopathological events in the gut mucosa initiated by severe CD4+T cell depletion and damage to the integrity of the intestinal epithelium including enterocyte apoptosis and tight junction disruption [75]. Increased levels of LPS have mainly been reported during later time points in HIV-infection [76], [77]. We did not observe significant correlations between microbial translocation marker levels and the frequency of mucosal Th17 cells (S2 Figure), and thus, we hypothesize that in early AHI systemic markers of microbial translocation, such as LPS, may be lower in plasma due to the host's functional pathogen clearance mechanisms (i.e. functional PMNs and macrophages, EndoCAB antibodies, etc.).

The extent to which the loss of Th17 cells causes dysregulation of mucosal immunity in HIV is still unclear, however Th17 cells are vital in maintaining a healthy mucosa and their loss is clearly detrimental. We show that a dramatic loss of mucosal Th17 and their functional capabilities occurs even earlier during AHI than previously described and that initiation of ART in Fiebig I/II, before this loss occurs, prevented alterations in Th17 numbers and functionality. Moreover, despite a partial reconstitution of Th17 cells numbers under ART initiated in Fiebig III, near the peak of viremia, polyfunctionality was not restored. The association of these events with local and systemic immune activation and its full reversion under very early initiated ART emphasizes the importance of strategies to prevent mucosal Th17 function loss and argues for early and aggressive intervention for therapeutic benefit and a potential functional cure [3], [4]. Our study provided evidence that identifying and treating AHI subjects is feasible [7], [72]. Future studies will be necessary to address evolution of mucosal integrity dysfunction during AHI as well as the long-term success of early initiation of ART as our observations are limited to 6 months of therapy.

## Materials and Methods

### Study subjects

The RV254/SEARCH 010 study is an ongoing prospective, open-label study in Bangkok, Thailand (clinicaltrials.gov NCT00796146). Samples from subjects who had VCT for HIV at The Thai Red Cross Anonymous Clinic and at the Silom Community Clinic were screened in real-time by pooled NAT and sequential EIA according to published methods [78]. Thai subjects who met the AHI laboratory criteria for Fiebig stages I to V [6] were enrolled and had clinical and laboratory assessments as previously described, including CD4, HIV RNA, liver transaminases, creatinine, lipids and urinalysis [33]. Plasma and peripheral blood mononuclear cells (PBMC) were cryopreserved. Sampling of gut-associated lymphoid tissue (GALT) occurred by sigmoid biopsy as an optional study procedure at baseline and 6 months. Biopsy pieces were either cryopreserved, embedded in paraffin or mucosal mononuclear cells (MMC) were isolated. Initiation of ART was voluntary and done as part of the enrollment in an accompanying protocol (clinicaltrials.gov NCT00796263). Treatment was initiated on average 3 days (range 0–5 days) from enrollment. The first 7 subjects included in this analysis were treated with standard doses of tenofovir/emtricitabine/raltegravir/maraviroc while the subsequent subjects were randomized to either this regimen or tenofovir/emtricitabine/efavirenz. Plasma, PBMC and MMC from HIV-uninfected and chronically HIV-infected (CHI) Thai volunteers were obtained from another protocol (clinicaltrials.gov NCT01397669) and were subject to the same procedures.

### Ethics statement

The RV254/SEARCH 010 study (clinicaltrials.gov NCT00796146) was approved by the Institutional Review Boards (IRBs) of Chulalongkorn University in Thailand and the Walter Reed Army Institute of Research in the United States. The protocol enrolling HIV-uninfected and chronically HIV-infected (CHI) Thai volunteers (clinicaltrials.gov NCT01397669) was approved by the Chulalongkorn University IRB. Initiation of ART was voluntary and done as part of the enrollment in an accompanying protocol (clinicaltrials.gov NCT00796263), approved by Chulalongkorn University IRB. For all studies mentioned above, subjects gave written informed consent.

### Diagnosis of acute HIV infection (AHI)

Diagnosis of AHI was performed as described previously [33]. In brief, AHI subjects were enrolled if they were Thai and fulfilled laboratory criteria for Fiebig stages I to V as follows: FI - positive HIV RNA, negative p24 antigen, non-reactive 3rd generation EIA; FII – positive HIV RNA, positive p24 antigen, non-reactive 3rd generation EIA; FIII - positive HIV RNA, positive p24 antigen, reactive 3rd generation EIA, negative western blot; FIV - positive HIV RNA, positive or negative p24 antigen, reactive 3rd generation EIA, indeterminate western blot; FV - positive HIV RNA, positive p24 antigen, reactive 3rd generation EIA, positive western blot except p31. All subjects had to have a non-reactive EIA by non-IgM sensitive EIA. The corresponding mean cumulative durations from the first detectable HIV RNA are 5 (FI), 10.3 (FII), 13.5 (FIII), 19.1 (FIV) and 88.6 (FV) days [6].

### Biopsy processing

Subjects underwent a routine sigmoidoscopy procedure under moderate conscious sedation. Approximately 30 endoscopic biopsies were randomly collected from the sigmoid colon using Radial Jaw 3 biopsy forceps (Boston Scientific, Natick, MA, USA) with 20–25 processed for flow cytometry analysis within 30 min of collection. In groups of five the biopsies were weighed and placed in 500 µl of RPMI media containing 10% human AB serum (HAB; Gemini Bio-Product, West Sacramento, CA, USA), 1% HEPES, 1% L-Glutamine, 0.1% Gentamicin (Invitrogen, Carlsbad, CA, USA), 1% Penicillin/Streptomycin and 2.5 µg/ml Amphotericin B (Invitrogen, Carlsbad, CA, USA). Samples were then digested using 0.5 mg/ml Collagenase II (Sigma, St. Louis, MO, USA). After digestion samples were filtered through a cell strainer, using a syringe with a 16-gauge blunt end needle. This procedure was repeated once or twice in case undigested tissue remained. After being washed twice with RPMI containing 1% HEPES, 1% L-Glutamin, 1% Penicillin/Streptomycin, 0.1% Gentamycin and 2.5 µg/ml Amphotericin B, MMC were counted and viable cell enumeration was determined using Trypan Blue exclusion and Beckman Coulter AcT5 hematology analyzer (Beckman Coulter, Fullerton, CA). Freshly isolated MMC were used for flow cytometry analysis. Subjects were screened incidental histopathology. For HIV RNA quantification biopsy pieces were collected in phosphate buffered saline (PBS) and subsequently stored in 1mL of RNAlater (Ambion, Foster, CA, USA) at −80°C.

### HIV RNA quantification

HIV RNA in plasma was measured using Roche Amplicor v 1.5 ultrasensitive assay with a lower quantification limit of 50 copies/mL (Roche Diagnostics, Branchburg, NJ, USA). For gut tissue, one to two biopsy pieces frozen in RNAlater (Ambion, Foster, CA, USA) were weighed then homogenized in AVL buffer (QIAamp Viral mini kit Cat No. 52,904, Netherlands) using a mini mortar and pestle. Extraction was completed per kit instructions. The Siemens Quantiplex HIV-1 3.0 assay was used to measure HIV-1 RNA copy number. Results are expressed as copies/mg of tissue.

### HIV-1 CRF01_ A/E lineage specific in situ hybridization, immunohistochemistry and quantitative image analysis

To ensure optimal detection of productively infected cells from HIV-infected subjects from Thailand, we designed a new set of HIV-1 CRF01_A/E lineage specific *in situ* hybridization riboprobes for these experiments. HIV-1 CRF01_A/E riboprobes were generated by PCR-based cloning of target regions from the full-length infectious molecular clone pCM235 from Thailand (Accession number AF259954) kindly provided by Dr. George Shaw (University of Pennsylvania). Riboprobes were generated targeting Gag (1454–1958), Pol (3998–4570), Accessory gene Vif/Vpr/Vpu/Tat/Rev/Env (5287–5825), Env (7836–8403) and Nef (8822–9122) using primers with either phage T3 (sense) or T7 (anti-sense) promoter sequences cloned upstream of the viral sequence and pooled into a cocktail at equal concentrations. HIV-1 *in situ* hybridization was performed as previously described [79]. HIV vRNA+ cells are stained blue/black and tissues are counterstained with nuclear fast red. Immunohistochemistry was performed using a biotin-free polymer approach (Golden Bridge International, Inc.) on 5-µm tissue sections mounted on glass slides, which were dewaxed and rehydrated with double-distilled H_2_O. Heat induced epitope retrieval (HIER) was performed by heating sections in 0.01% citraconic anhydride containing 0.05% Tween-20 in a pressure cooker set at 122–125°C for 30 seconds. Slides were incubated with blocking buffer (TBS with 0.05% Tween-20 and 0.25% casein) for 10 minutes and then incubated with mouse anti-CD68 (1∶400; clone KP1, Dako), mouse anti-CD163 (1∶400; clone 10D6; Novocastra/Leica) and rabbit monoclonal anti-CD4 (1∶200; clone EPR6855; Abcam, Inc.) diluted in blocking buffer overnight at 4°C. Slides were washed in 1x TBS with 0.05% Tween-20 and endogenous peroxidases blocked using 1.5% (v/v) H_2_O_2_ in TBS (pH 7.4) for 10 minutes. Slides were incubated with Mouse Polink-1 AP for 10 minutes followed by Rabbit Polink-1 HRP for 30 minutes at room temperature. Sections were first incubated with Impact DAB (3,3′-diaminobenzidine; Vector Laboratories) to develop the CD4, washed and developed with Warp Red (Biocare Medical, Inc.) to mask the faint CD4 expressed on APCs allowing for specific identification of CD4+T cells. Slides were washed in ddH_2_O, counterstained with hematoxylin, mounted in Permount (Fisher Scientific), and scanned at high magnification (x200) using the ScanScope CS System (Aperio Technologies) yielding high-resolution data from the entire tissue section. Representative regions of interest (ROIs; 500 mm^2^) were identified and high-resolution images extracted from these whole-tissue scans. The percent area of the lamina propria that stained for CD4+T cells (excluding APC CD4) were quantified using Photoshop CS5 and Fovea tools.

### Immunophenotyping of PBMC and sigmoid colon MMC

Immunophenotyping was performed on cryopreserved PBMC and freshly isolated MMC from sigmoid colon. Cells were first stained with Aqua Live/Dead dye (Invitrogen, Eugene, Oregon, USA). Subsequently samples were stained with the following antibodies to identify the different cell subsets. Regulatory T cells (Treg) were stained with anti-CD3 PE-Cy7 (Invitrogen, Eugene, Oregon, USA), anti-CD4 ECD (Beckman Coulter, Brea, CA, USA), anti-CD8 PerCP-Cy5.5 (BD Bioscience, San Jose, CA, USA) and anti-CD25 APC-Cy7 (BD Pharmingen, San Diego, CA, USA) for 20 min at room temperature. Subsequently cells were washed twice with Permeabilization Buffer provided in the FoxP3 Staining Buffer Set and stained with anti-FoxP3 APC (eBioscience, San Diego, CA, USA) and anti-Ki67 FITC (BD Pharmingen, San Diego, CA, USA) for 30 min at 4°C. CD4+ and CD8+ memory T cells were defined using anti-CD4-QDot605, anti-CD3-PE-TexasRed (Invitrogen, Eugene, Oregon, USA), anti-CD8-V450 (BD Horizon, San Diego, CA, USA), anti-CD27-AlexaFluor700 and anti-CD45RO-PE-Cy7 (BD Pharmingen, San Diego, CA, USA) for 20 min at room temperature. Subsequently cells were washed with PBS and stained with anti-CCR5-APC-Cy7 (BD Pharmingen, San Diego, CA, USA) for 30 min at 37°C. Activation status of CD4+ and CD8+T cells was determined by staining cells using anti-CD3 PE-Cy7 (Invitrogen, Eugene, Oregon, USA), anti-CD4 ECD (Beckman Coulter, Brea, CA, USA), anti-CD8 PerCP-Cy5.5, anti-HLA-DR V450 and anti-CD38 APC (BD Bioscience, San Jose, CA, USA) for 20 min at room temperature, subsequently washed with Permeabilization Buffer and stained with anti-Ki67 FITC (eBioscience, San Diego, CA, USA) for 30 min at 4°C. Post staining cells were resuspended in 1% Formaldehyde and acquired within 24 hours using a custom built BD LSRII or Fortessa flow cytometer (BD, San Jose, CA, USA) and analyzed using FlowJo software version 9.6.3 or higher (TreeStar, Ashland, OR, USA). At least 80,000 live cells were acquired in the lymphocyte gate. In the peripheral blood percentage and absolute numbers of CD4+ and CD8+T cells were determined using the BD Multitest IMK Kit (BD Bioscience, San Jose, CA, USA).

### Quantification of cytokine expression

Cryopreserved PBMC and freshly isolated MMC were rested over night at 37°C before stimulation for 5 hours with 40 ng/mL of Phorbolmyristate acetate (PMA) and 1 µM Ionomycin (Sigma-Aldrich, St. Louis, MO, USA) in the presence of 1 µl/mL of Brefeldin A (BD Bioscience, San Jose, CA, USA) to prevent cytokine release. As negative control cells remained unstimulated with 1 µl/mL Brefeldin A added for 5 hours of incubation. After two washes with RPMI medium containing 10% heat-inactivated HAB serum, cells were fixed and permeabilized according to the BD Cytofix/Cytoperm protocol. Subsequently cells were stained with the following antibodies: anti-CD3 PE-Cy7 (Invitrogen, Eugene, Oregon, USA), anti-CD4 ECD (Beckman Coulter, Brea, CA, USA), anti-IL-2 FITC, anti-CD8 PerCP-Cy5.5 (BD Bioscience, San Jose, CA, USA), anti-IL17A PE (BD Pharmingen, San Diego, CA, USA), anti-IL22 APC (R&D systems, Minneapolis, MN, USA) and IFNγ eFlour450 (eBioscience, San Diego, CA, USA) for 20 min at 4°C. Samples were acquired and analyzed as described above. Positive responses shown were calculated after unstimulated background was subtracted. Frequencies of IFNγ, IL-2 and IL-17 and/or IL-22 expressing cells is based on the population of CD4+T cells, while the frequency of triple-cytokine producing cells (IFNγ, IL-2 and IL-22) is based on the population of CD4+IL-17+T cells.

### Calculation of absolute numbers of colonic T-cell subsets

Absolute numbers of CD4+ and CD8+ T-cell subsets per gram of gut tissue were calculated by multiplying the total viable cell count by percentages obtained from flow cytometry analysis. The total cell count per gram of tissue was calculated by dividing the viable cell count by the tissue weight. This proportion was then multiplied by the percent of cells in the live lymphocyte gate and that number was subsequently multiplied by the percent of CD3+ lymphocytes. The absolute number of colonic CD3+T cells was used in conjunction with the subset percentages to determine the absolute number of each T-cell subset per gram of biopsy tissue.

### Plasma levels of cytokines and biomarkers

Cytokines and biomarkers were measured using cryopreserved EDTA plasma. Interferon gamma-induced protein (IP)-10 was measured by standard ELISA (Invitrogen, Carlsbad CA). D-dimer was measured using an enzyme-linked fluorescent assay on a VIDAS instrument (bioMerieux Inc., Durham, North Carolina, USA). Hyaluronic acid (HA) was measured using a commercially available HA test kits (Corgenix, Inc, Westminster, Colorado, USA). C-Reactive Protein (CRP) was measured by electro-chemo-luminescence (Meso Scale Discovery, Gaithersburg, Maryland, USA). Soluble CD14 (sCD14) was measured in duplicates using a commercially available ELISA assay (R&D Systems, Minneapolis, MN, USA), and analyzed according to manufacturer's protocol. TNFα was quantified from citrate plasma in triplicate by custom ELISA array according to the manufacturer's protocol (Quansys Biosciences, Logan UT). Data was captured on the Odyssey infrared imaging system (Li-Cor Biosciences, Lincoln, NE) and analyzed using Quansys Q-view Plus software (Quansys Biosciences). Intestinal fatty acid binding protein (I-FABP) was measured using a commercially available ELISA assay (R&D Systems, Minneapolis, MN, USA) with samples diluted to 10% in sCD14 diluent. Lipopolysaccharide (LPS) levels were quantified by first diluting fasting plasma samples, collected in EDTA tubes, to 10% with endotoxin-free water and subsequent heat inactivation of plasma proteins for 15 minutes at 80°C. Measurements of the samples were made with a Limulus Amebocyte Assay (Lonza Group Ltd., Switzerland). Samples were measured in duplicate, background was subtracted and LPS levels were calculated by first setting the y-intercept for the standard regression line at zero and then by the manufacturer's protocol.

### Statistical analysis

Due to low subject numbers in certain Fiebig stages, such as FII (n = 4), FIV (n = 1) and FV (n = 3), FI and II and FIV and V were combined into two groups: FI/II and FIV/V, respectively for the analysis. The Mann-Whitney U test was used for between group comparisons and Spearman's rank test was used to evaluate associations. All values reported were median (interquartile range). Statistical tests were 2-sided with p-values <0.05 considered statistically significant. Statistical analyses were performed using Prism version 6.0b software program (GraphPad Software, Inc., La Jolla, CA, USA).

## Supporting Information

S1 Figure Frequency of mucosal T_reg_ and cycling T_reg_ expressing Ki67. (**a**) Frequency of mucosal T_reg_ remained unchanged with progression of Fiebig stage, while there was a significant increase observed in CHI compared to HIV- and AHI subjects (p<0.001). (**b**) Frequency of cycling mucosal T_reg_ (Ki67+) increased significantly with progression of Fiebig stage. *p≤0.05 and ***p≤0.001; FI (black circle), FII (red circle), FIV (red square), FV (black square).
(TIF)

S2 Figure Correlation between the frequency of IL-17 expressing mucosal CD4+ T cells and plasma levels of systemic activation markers. Shown are the correlation between mucosal IL-17 expressing CD4+ T cells and (**a**) C-reactive protein (CRP), (**b**) Hyaluronic Acid (HA), (**c**) Tumor Necrosis Factor-alpha (TNFα), (**d**) Interferon-inducible protein-10 (IP-10), (**e**) Lipopolysaccharide (LPS), (**f**) soluble CD14 (sCD14) and (**g**) D-dimer; FI (black circle), FII (red circle), FIII (black triangle), FIV (red square), FV (black square).
(TIFF)

S1 Table Laboratory stages of primary HIV infection based on nucleic acid testing and HIV serological markers.
(DOCX)

S2 Table Results of Spearman rank tests comparing the percentage of activated (%HLA-DR+/CD38+) and cycling (Ki67+) CD4+ and CD8+ T cells in the peripheral blood and the sigmoid colon with frequency of CD4+ T cells and HIV RNA viral load in the respective compartment among AHI subjects (FI/II, FII and FIV/V).
(DOCX)

S3 Table Proportion of mucosal and peripheral blood cell subsets before and after 6 month of ART for FI/II and FIII subjects.
(DOCX)

S4 Table P-values comparing the proportion of mucosal and peripheral blood cell subsets displayed in Table S3 for FI/II and FIII subjects before and after 6 month of ART compared to HIV- subjects.
(DOCX)

S5 Table P-values comparing the proportion of mucosal and peripheral blood cell subsets displayed in Table S3 before and after 6 months of ART for FI/II and FIII subjects.
(DOCX)
