# Supplementary figures and images for: Initiation of ART during Early Acute HIV Infection Preserves Mucosal Th17 Function and Reverses HIV-Related Immune Activation
Source: PLoS Pathog. 2014 Dec 11;10(12):e1004543. doi: 10.1371/journal.ppat.1004543 (PMC4263756; doi:10.1371/journal.ppat.1004543)

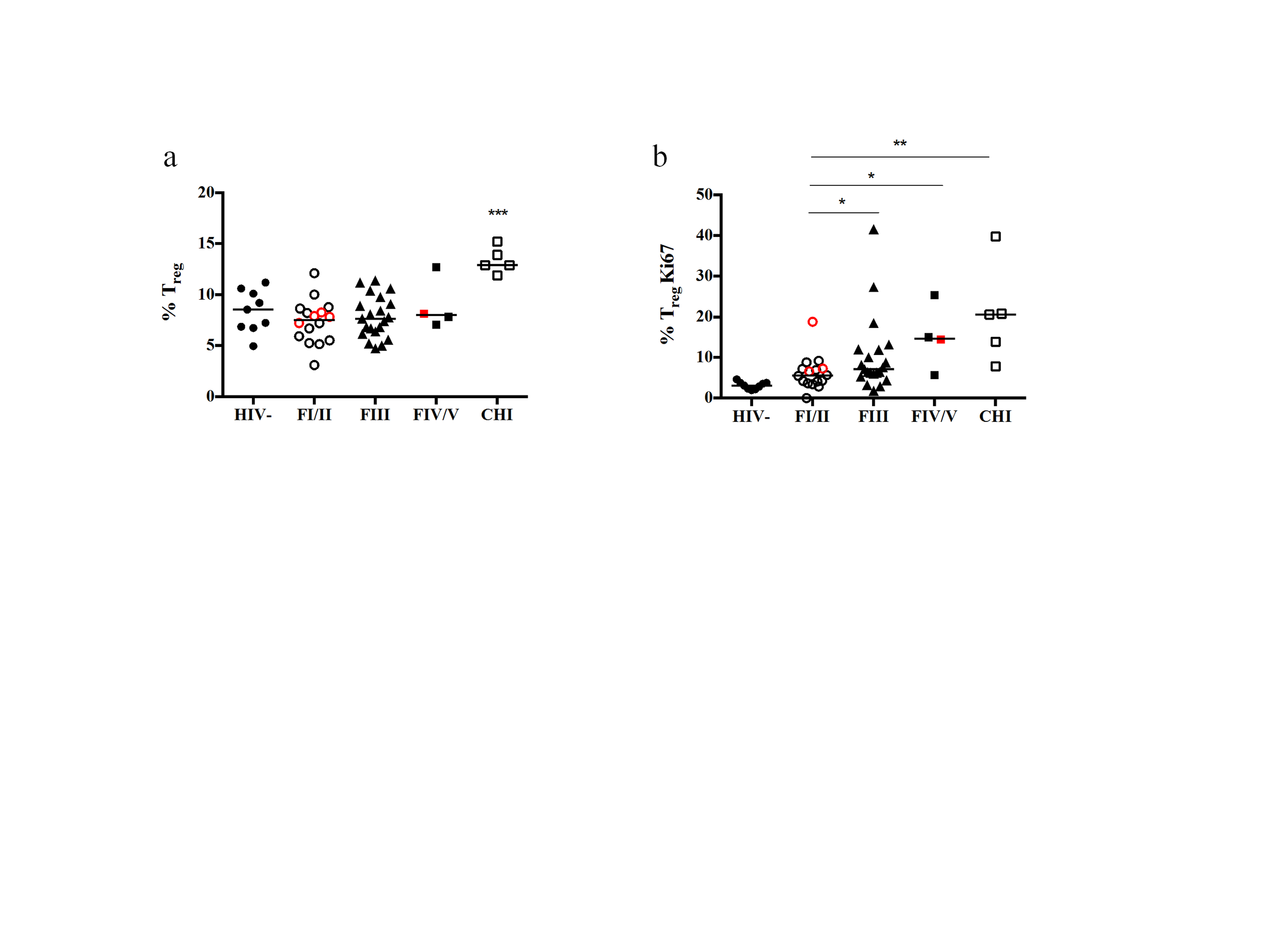

Supplement: S1 Figure — Frequency of mucosal Treg and cycling Treg expressing Ki67. (a) Frequency of mucosal Treg remained unchanged with progression of Fiebig stage, while there was a significant increase observed in CHI compared to HIV- and AHI subjects (p<0.001). (b) Frequency of cycling mucosal Treg (Ki67+) increased significantly with progression of Fiebig stage. *p≤0.05 and ***p≤0.001; FI (black circle), FII (red circle), FIV (red square), FV (black square). (TIF) [file ppat.1004543.s001.tif]

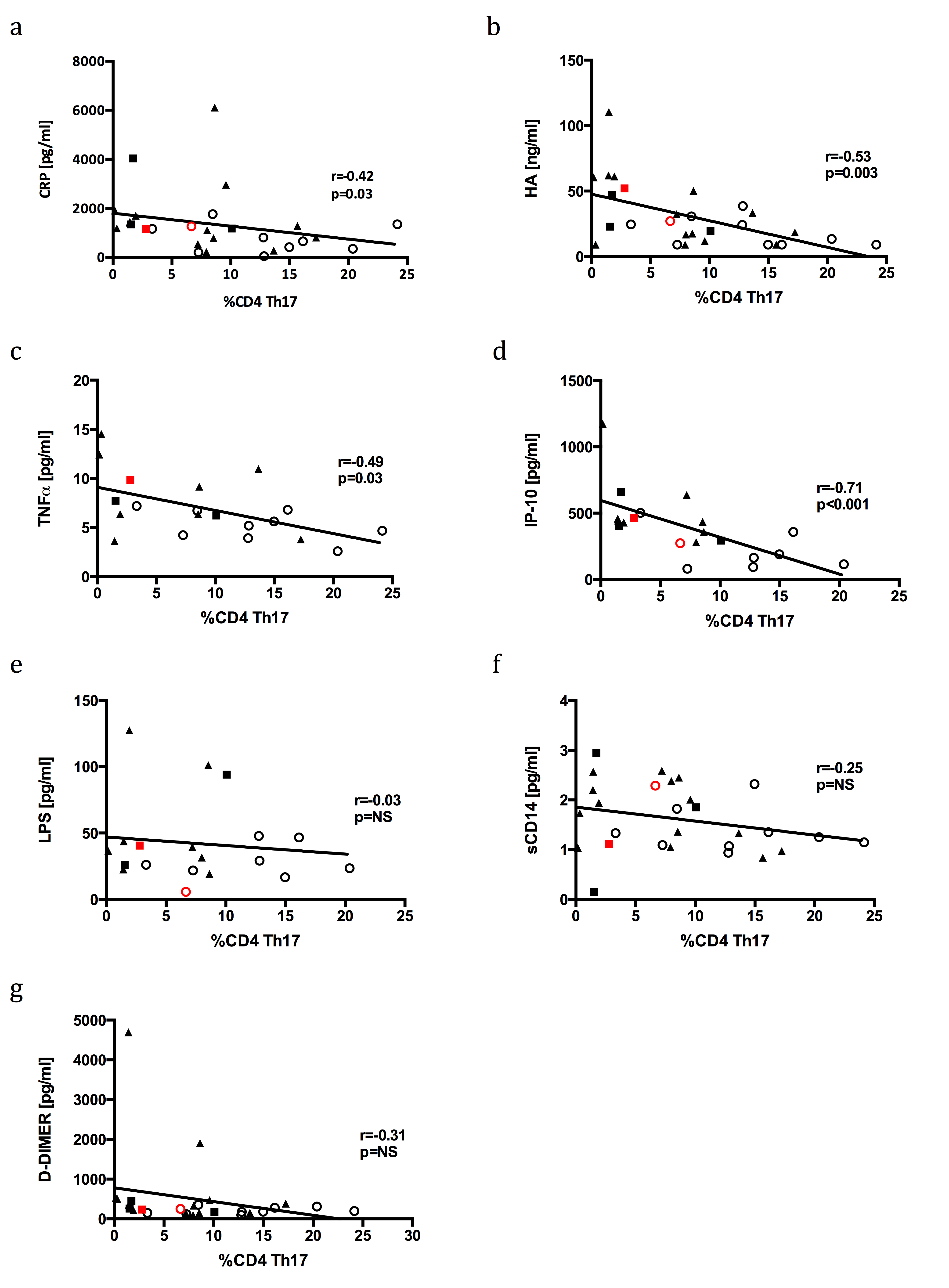

Supplement: S2 Figure — Correlation between the frequency of IL-17 expressing mucosal CD4+ T cells and plasma levels of systemic activation markers. Shown are the correlation between mucosal IL-17 expressing CD4+ T cells and (a) C-reactive protein (CRP), (b) Hyaluronic Acid (HA), (c) Tumor Necrosis Factor-alpha (TNFα), (d) Interferon-inducible protein-10 (IP-10), (e) Lipopolysaccharide (LPS), (f) soluble CD14 (sCD14) and (g) D-dimer; FI (black circle), FII (red circle), FIII (black triangle), FIV (red square), FV (black square). (TIFF) [file ppat.1004543.s002.tiff]
