# Supplementary material for: Initiation of ART during Early Acute HIV Infection Preserves Mucosal Th17 Function and Reverses HIV-Related Immune Activation
Source: PLoS Pathog. 2014 Dec 11;10(12):e1004543. doi: 10.1371/journal.ppat.1004543 (PMC4263756; doi:10.1371/journal.ppat.1004543)
Supplement: S1 Table — Laboratory stages of primary HIV infection based on nucleic acid testing and HIV serological markers. (DOCX) [file ppat.1004543.s003.docx]

| **Patient** | **Fiebig Stage** | **RNA** | **p24 Antigen** | **3^rd^ Gen EIA** | **Western Blot** |
| --- | --- | --- | --- | --- | --- |
|  |  |  |  |  |  |
| 01 | III | + | + | + | - |
| 02 | II | + | + | - | - |
| 03 | III | + | + | + | - |
| 04 | I | + | + | - | - |
| 05 | II | + | + | - | - |
| 06 | III | + | + | + | - |
| 07 | III | + | - | + | - |
| 08 | III | + | + | + | - |
| 09 | I | + | - | - | - |
| 10 | III | + | + | + | - |
| 11 | I | + | - | - | - |
| 12 | III | + | + | + | - |
| 13 | III | + | + | + | - |
| 14 | II | + | + | - | - |
| 15 | III | + | - | + | - |
| 16 | V | + | + | + | + |
| 17 | V | + | + | + | + |
| 18 | III | + | + | + | - |
| 19 | III | + | + | + | - |
| 20 | III | + | + | + | - |
| 21 | I | + | - | - | - |
| 22 | I | + | - | - | - |
| 23 | II | + | + | - | - |
| 24 | III | + | - | + | - |
| 25 | III | + | + | + | - |
| 26 | V | + | + | + | + |
| 27 | I | + | - | - | - |
| 28 | III | + | + | + | - |
| 29 | IV | + | + | + | - |
| 30 | I | + | - | - | - |
| 31 | I | + | - | - | - |
| 32 | I | + | - | - | - |
| 33 | III | + | - | + | - |
| 34 | I | + | - | - | - |
| 35 | I | + | - | - | - |
| 36 | III | + | - | + | - |
| 37 | I | + | - | - | - |
| 38 | III | + | + | + | - |
| 39 | III | + | - | + | - |
| 40 | III | + | + | + | - |
| 41 | I | + | - | - | - |
| 42 | III | + | - | + | - |
|  |  |  |  |  |  |
