# Supplementary material for: Initiation of ART during Early Acute HIV Infection Preserves Mucosal Th17 Function and Reverses HIV-Related Immune Activation
Source: PLoS Pathog. 2014 Dec 11;10(12):e1004543. doi: 10.1371/journal.ppat.1004543 (PMC4263756; doi:10.1371/journal.ppat.1004543)
Supplement: S2 Table — Results of Spearman rank tests comparing the percentage of activated (%HLA-DR+/CD38+) and cycling (Ki67+) CD4+ and CD8+ T cells in the peripheral blood and the sigmoid colon with frequency of CD4+ T cells and HIV RNA viral load in the respective compartment among AHI subjects (FI/II, FII and FIV/V). (DOCX) [file ppat.1004543.s004.docx]

|  | **%CD4 (PB)** | **%CD4 (Colon)** |
| --- | --- | --- |
| %CD4 HLA-DR/CD38  (at matching site) | -0.33  **0.03** | -0.04  0.80 |
| %CD8 HLA-DR/CD38  (at matching site) | -0.23  0.15 | -0.42  **0.007** |
|  | **Plasma HIV RNA**  **(copies per ml)** | **Colonic HIV RNA**  **(copies per mg)** |
| %CD4 HLA-DR/CD38  (PB) | 0.27  0.07 | 0.21  0.20 |
| %CD4 HLA-DR/CD38  (Colon) | 0.16  0.33 | 0.03  0.87 |
| %CD8 HLA-DR/CD38  (PB) | 0.27  0.09 | 0.41  **0.01** |
| %CD8 HLA-DR/CD38  (Colon) | 0.51  **<0.001** | 0.38  **0.02** |
|  | **%CD4 (PB)** | **%CD4 (Colon)** |
| %CD4 Ki67  (at matching site) | -0.38  **0.01** | -0.43  **0.006** |
| %CD8 Ki67  (at matching site) | -0.59  **<0.001** | -0.54  **<0.001** |
|  | **Plasma HIV RNA**  **(copies per ml)** | **Colonic HIV RNA**  **(copies per mg)** |
| %CD4 Ki67  (PB) | -0.007  0.64 | 0.05  0.76 |
| %CD4 Ki67  (Colon) | 0.34  **0.03** | 0.41  **0.009** |
| %CD8 Ki67  (PB) | 0.39  **0.01** | 0.52  **<0.001** |
| %CD8 Ki67  (Colon) | 0.56  **<0.001** | 0.53  **<0.001** |
