# Supplementary material for: Initiation of ART during Early Acute HIV Infection Preserves Mucosal Th17 Function and Reverses HIV-Related Immune Activation
Source: PLoS Pathog. 2014 Dec 11;10(12):e1004543. doi: 10.1371/journal.ppat.1004543 (PMC4263756; doi:10.1371/journal.ppat.1004543)
Supplement: S3 Table — Proportion of mucosal and peripheral blood cell subsets before and after 6 month of ART for FI/II and FIII subjects. (DOCX) [file ppat.1004543.s005.docx]

|  | HIV- (n=9) | FI/II | | FIII | |
| --- | --- | --- | --- | --- | --- |
|  |  | pre-ART (n=17) | post-ART (n=14)^+^ | pre-ART (n=21) | post-ART (n=15)^+^ |
| sigmoid colon |  |  |  |  |  |
| % CD4 | 56.1 (48.9, 61.1) | 49.8 (46.4, 58.1) | 46.5 (28.8, 58.1) | 35.2 (23.8, 43.1) | 35.9 (18.2, 59.8) |
| %CD4+CCR5+ | 69.1 (66.5, 70.6) | 67.3 (50.9, 74.4) | 62.5 (11.5, 80.6) | 35.5 (10.8, 57.5) | 54.4 (10.8, 75.6) |
| % IL-17 | 13.5 (8.4, 16.9) | 12.8 (3.3, 24.1) | 13.2 (1, 22.5) | 7.9 (0.2, 17.2) | 10.2 (0.9, 19.1) |
| % IL22 | 3.6 (0.3, 6.5) | 2.9 (0.2, 8.7) | 3.2 (1.1, 30.2) | 1.3 (0.1, 7.1) | 1.2 (0.2, 6.6) |
| % IL-17/IL-22 | 4.0 (2.4, 5.8) | 3.7 (0.9, 7.3) | 3.6 (0.1, 12.3) | 1.7 (0, 5.1) | 1.5 (0, 4) |
| % CD8 DR+CD38+ | 1.9 (0.9, 1.9) | 4.4 (1.1, 24.8) | 2.1 (0.7, 3.8) | 8.9 (3.8, 40.2) | 5.0 (1.5, 8.8) |
| peripheral blood |  |  |  |  |  |
| % CD8 DR+CD38+ | 3.0 (0.1, 2.9) | 7.8 (1.9, 20.5) | 3.7 (1.6, 12.6) | 15 (8.3, 54.4) | 9.0 (1.3, 15.7) |

All data are median (interquartile rang); ^+^Th17 data are only available for a subset of samples due to cell availability; DR: HLA-DR
