# Supplementary material for: Initiation of ART during Early Acute HIV Infection Preserves Mucosal Th17 Function and Reverses HIV-Related Immune Activation
Source: PLoS Pathog. 2014 Dec 11;10(12):e1004543. doi: 10.1371/journal.ppat.1004543 (PMC4263756; doi:10.1371/journal.ppat.1004543)
Supplement: S4 Table — P-values comparing the proportion of mucosal and peripheral blood cell subsets displayed in Table S3 for FI/II and FIII subjects before and after 6 month of ART compared to HIV- subjects. (DOCX) [file ppat.1004543.s006.docx]

|  | HIV- comparing to: | | | |
| --- | --- | --- | --- | --- |
|  | FI/II pre-ART | FI/II post-ART | FIII pre-ART | FIII post-ART |
| sigmoid colon |  |  |  |  |
| % CD4 | NS | 0.05 | <0.001 | 0.002 |
| %CD4+CCR5+ | NS | NS | <0.001 | 0.02 |
| % IL-17 | NS | NS | 0.01 | NS |
| % IL22 | NS | NS | 0.03 | 0.05 |
| % IL-17/IL-22 | NS | NS | 0.02 | 0.003 |
| % CD8 DR+CD38+ | <0.001 | NS | <0.001 | <0.001 |
| peripheral blood |  |  |  |  |
| % CD8 DR+CD38+ | <0.001 | NS | <0.001 | <0.001 |

All comparisons were made to HIV-; DR: HLA-DR
