# Supplementary material for: Initiation of ART during Early Acute HIV Infection Preserves Mucosal Th17 Function and Reverses HIV-Related Immune Activation
Source: PLoS Pathog. 2014 Dec 11;10(12):e1004543. doi: 10.1371/journal.ppat.1004543 (PMC4263756; doi:10.1371/journal.ppat.1004543)
Supplement: S5 Table — P-values comparing the proportion of mucosal and peripheral blood cell subsets displayed in Table S3 before and after 6 months of ART for FI/II and FIII subjects. (DOCX) [file ppat.1004543.s007.docx]

|  | FI/II^*^ | FIII^*^ | post-ART^**^ |
| --- | --- | --- | --- |
| sigmoid colon |  |  |  |
| % CD4 | NS | NS | 0.03 |
| %CD4+CCR5+ | NS | 0.02 | NS |
| % IL-17 | NS | 0.05 | NS |
| % IL22 | NS | NS | 0.05 |
| % IL-17/IL-22 | NS | NS | 0.04 |
| % CD8 DR+CD38+ | 0.001 | <0.001 | <0.001 |
| peripheral blood |  |  |  |
| % CD8 DR+CD38+ | 0.007 | 0.003 | 0.03 |

*Comparisons were made between pre- and post-ART for FI/II and FIII; **Comparisons were made between FI/II and FIII

post-ART; DR: HLA-DR
